# Exploring gene-patient association to identify personalized cancer driver genes by linear neighborhood propagation

**DOI:** 10.1186/s12859-024-05662-4

**Published:** 2024-01-22

**Authors:** Yiran Huang, Fuhao Chen, Hongtao Sun, Cheng Zhong

**Affiliations:** 1 School of Computer, Electronics and Information, Guangxi University, Nanning, 530004 China; 2 Key Laboratory of Parallel, Distributed and Intelligent Computing in Guangxi Universities and Colleges, Guangxi University, Nanning, 530004 China; 3 Guangxi Key Laboratory of Multimedia Communications and Network Technology, Guangxi University, Nanning, 530004 China

**Keywords:** Personalized cancer driver gene, Gene-patient association, Linear neighborhood propagation, Gene interaction network

## Abstract

**Background:**

Driver genes play a vital role in the development of cancer. Identifying driver genes is critical for diagnosing and understanding cancer. However, challenges remain in identifying personalized driver genes due to tumor heterogeneity of cancer. Although many computational methods have been developed to solve this problem, few efforts have been undertaken to explore gene-patient associations to identify personalized driver genes.

**Results:**

Here we propose a method called LPDriver to identify personalized cancer driver genes by employing linear neighborhood propagation model on individual genetic data. LPDriver builds personalized gene network based on the genetic data of individual patients, extracts the gene-patient associations from the bipartite graph of the personalized gene network and utilizes a linear neighborhood propagation model to mine gene-patient associations to detect personalized driver genes. The experimental results demonstrate that as compared to the existing methods, our method shows competitive performance and can predict cancer driver genes in a more accurate way. Furthermore, these results also show that besides revealing novel driver genes that have been reported to be related with cancer, LPDriver is also able to identify personalized cancer driver genes for individual patients by their network characteristics even if the mutation data of genes are hidden.

**Conclusions:**

LPDriver can provide an effective approach to predict personalized cancer driver genes, which could promote the diagnosis and treatment of cancer. The source code and data are freely available at https://github.com/hyr0771/LPDriver.

**Supplementary Information:**

The online version contains supplementary material available at 10.1186/s12859-024-05662-4.

## Background

Cancer is a heterogeneous disease driven by genetic alterations [1]. Identifying the cancer driver genes with alterations plays a crucial role in the treatment and diagnosis of cancer [2–5]. A number of computational methods have been proposed to identify cancer driver genes in recent years [2]. Most of these methods concentrate on identifying driver genes in specific types or subtypes of cancer [3]. Based on the rationale, these computational methods can mainly be grouped in mutation-based methods and the network-based methods. Mutation-based methods [6–10] employ the characteristics of gene mutations to identify cancer driver genes while the network-based methods [11–14] utilize gene interaction networks to assess the role of genes to predict cancer driver genes.

Several mutation-based methods have been proposed to identify cancer driver genes, each with its unique approach and hypothesis [15]. MutSigCV [7] evaluates the gene mutation frequencies that exceed what is expected to identify potential cancer driver genes. However, it may incorrectly identify the genes with frequent mutations that are non-contributory to cancer development as potential cancer driver genes. Unlike MutSigCV, OncodriveFM [8] hypothesizes that the genes with significantly functional impact are more likely to be candidate driver genes. OncodriveFM evaluates the bias of gene mutations with functional impacts rather than the sheer mutation count, enabling the detection of driver genes with low recurrence but significant roles in cancer development. OncodriveFML [6], similar to OncodriveFM, employs functional impact assessment but extends its scope to both coding and non-coding mutations. DriverML [9] adopts a different approach by considering how mutation types affect the functional impact of mutations. It uses a supervised machine learning approach with pan-cancer training data to optimize weight parameters for mutation types, scoring functional influences of gene alterations. ActiveDriver [10], on the other hand, identifies cancer drivers based on the structural consequences of gene mutations, particularly focusing on the enrichment in post-translational modification sites like phosphorylation, acetylation, and ubiquitination sites. Although these mutation-based methods offer valuable insights into the identification of cancer driver genes, they face the problems of the incomplete gene mutation databases caused by cancer heterogeneity. This may limit their ability to comprehensively identify driver genes.

On the other hand, the network-based methods identify cancer driver genes by incorporating the information of pathways, gene–gene or protein–protein interactions to measure the gene roles in the biological networks [15–17]. For instance, CBNA [11] combines the network controllability analysis with mutation data, allowing it to pinpoint both coding and miRNA driver genes within gene networks. Meanwhile CBNA can also be employed to uncover the drivers specific to particular types or subtypes of cancer. In contrast to CBNA, DriverNet [12] integrates various multi-omics data such as gene expression data and biological pathway information to construct bipartite gene network, and utilizes greedy optimization search to identifies driver genes with high outlying expression in the bipartite gene network. Similarly, Subdyquency [13] integrates mutated genes’ variation frequency and its interactions with dysregulated genes in a certain compartment to build bipartite graph, then employs random walk method on the built graph to produce walking score for each mutated gene of patient to pinpoint candidate driver genes. Different from these methods, MEMo [14] (Mutual Exclusivity Modules) takes a module-centric approach, using mutual exclusivity techniques in biological networks to discover oncogenic network modules. MEMo suggests that genomic alterations in the same cancer type tend to occur within a limited number of pathways and are unlikely to coexist within the same patient. These abovementioned methods provide diverse strategies for uncovering cancer driver genes, contributing valuable insights into the molecular mechanisms of cancer.

Nevertheless, all these methods identify cancer driver genes at the population level. Due to tumor heterogeneity in cancer, different patients may have different genetic alterations and their tumors may be caused by different genes, and two patients who suffer from the same kind of cancer and receive the same remedy may have different prognosis [2, 3]. Thus, it is necessary to identify cancer drivers specific to an individual patient for personalized diagnosis and treatment [2, 3]. DawnRank [18] for the first time utilizes a ranking framework to assess the connectivity and the amount of differential expression genes in gene interaction network. By combining gene ranks with somatic alteration data, such as copy variation number, DawnRank effectively detects individual driver alterations. However, it relies on the same gene regulatory network for all patients, potentially missing patient-specific regulatory information. To address this limitation, SCS [19] constructs a personalized gene regulatory network for each patient using the gene expression data of patients and normal people. SCS identifies personalized cancer driver genes as the minimal set of the most differentially expressed genes in the constructed network. Further, PRODIGY [20] adopts Steiner tree model to evaluate the impact of the genes with mutations on the deregulated pathways to identify personalized cancer driver genes. Later, PersonaDrive [21] tries to construct a personalized bipartite graph that links mutated genes to differentially expressed genes for each patient, and calculates the edge weights of the graph based on the overlap between the mutated gene and the differentially expressed gene pair in biological pathways. Subsequently, it ranks the the potential driver genes based on their influence scores evaluated by the edge weights in the bipartite graph. Similarly, BetweenNET [22] combines patient genomic data with protein–protein interaction network to build customized gene interaction network and identifies personalized cancer driver genes in the customized network. Meanwhile, based on the structural controllability theory. Cheng et al. [23] proposed a weighted minimum dominating set network model WMDS.net to find the key regulators of gene co-expression networks to determine cancer driver genes. Distinguishing from these methods focusing on coding driver genes, Pham et al. [2] shifts the focus to the comprehensive exploration of coding and non-coding cancer drivers. They introduced a network-based approach named pDriver to identify personalized coding and miRNA cancer drivers. Recently, Guo et al. [24] proposed a structure-based network control method called PNC for identifying personalized cancer driver genes based on the network control method NCUA. In order to verify the effectiveness of NCUA, Guo et al. replaced NCUA with the state-of-the-art structure-based network control methods MMS [25] and MDS [25] in PNC respectively and compared their performance in identifying personalized cancer driver genes. The experimental comparison results showed that as compared to MMS and MDS, NCUA is more effective for PNC in identifying personalized cancer driver genes.

In this paper, we propose a novel method, called LPDriver, to identify personalized cancer driver genes. In comparison to existing methods for finding personalized cancer driver genes, LPDriver mainly includes the following advantages:

i. LPDriver attempts for the first time to explore the gene-patient associations extracted from personalized gene network to mine functionally similar genes among patients for identifying personalized driver genes. Nevertheless, LPDriver does not need to bind gene-patient associations with the known driver genes and the mutation genes to detect driver genes, which will promote and facilitate identification of cancer driver genes.
ii. Distinguishing from existing network-based methods, LPDriver finds the genes in the maximum matching set of the personalized gene network, which maximumly cover most but not all edges of the gene network, as potential driver genes. This could further extend exploration of driver genes.

To take advantages of the gene interaction network specific to a patient of a specific cancer, we first construct the personalized gene interaction network for each patient based on the tumor gene expression data of the patient. Next, we build the personalized gene network for the given patient upon the difference between the gene network built on all patients of the specific cancer dataset (e.g. the cancer dataset of The Cancer Genome Atlas) and the gene network built on all patients excluding the patient under consideration. Then we extract potential driver genes by finding the maximum matchings of the bipartite graph of the personalized gene network to build gene-patient associations. Finally, we utilize a linear neighborhood propagation model to mine the linear neighborhood similarity of genes among patients to infer the personalized driver genes from the built gene-patient associations.

We applied LPDriver on multiple cancer datasets of The Cancer Genome Atlas (TCGA) [26] and validated the results by considering the cancer driver genes in the Network of Cancer Genes (NCG) [27] and Cancer Genes Census (CGC) [28] as the benchmark. The experimental comparison results demonstrate that LPDriver is more effective than the existing methods in detecting cancer driver genes. Moreover, the experimental results also show that LPDriver not only can reveal personalized cancer driver genes for individual patient, but also detect some potentially novel driver genes that have been documented to be related to cancer. Generally, LPDriver is an effective and applicable complement to the existing methods for identifying cancer driver genes.

## Materials and methods

**Fig. 1:**
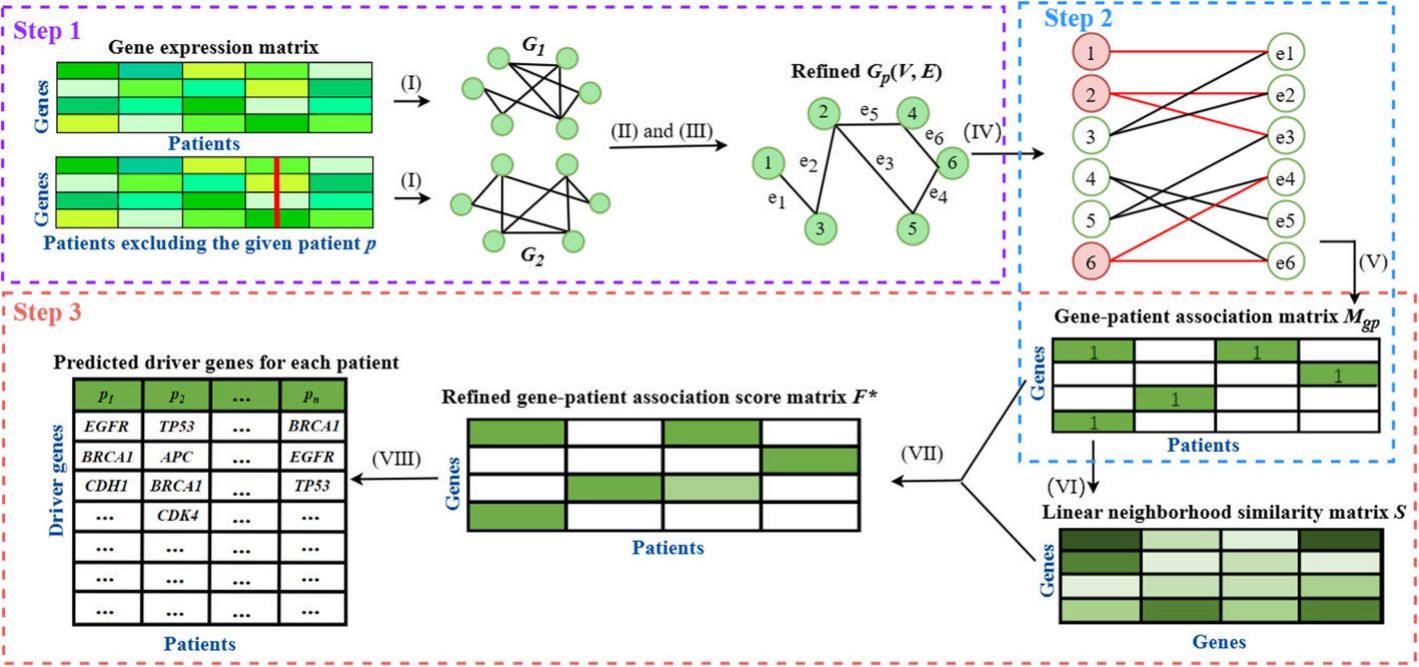
Illustration of LPDriver. **I** Constructing the gene interaction network *G*_*1*_ and *G*_*2*_. **II** Removing the edges that exist in both *G*_*1*_ and *G*_*2*_. **III** Removing the edges(interactions) that are not supported by the known gene interaction network. **IV** Transform *G*_*p*_*(V,E)* into bipartite graph and identify driver genes of patient *p* in bipartite graph. **V** Identify the potential driver genes of all patients to construct the matrix *M*_*gp*_. **VI** Compute the linear neighborhood similarity of genes among patients in *M*_*gp*_. **VII** Adopt label propagation method based on matrix *S* to refine *M*_*gp*_. **VIII** Identify highly rank genes as the personalized driver genes from F*

LPDriver identifies personalized cancer driver genes by three main steps: (1) Constructing personalized gene interaction network (PGIN) using the gene expression data of tumor samples of patients. (2) Identifying potential gene-patient associations by finding the maximum bipartite matchings from the bipartite graph of PGIN. (3) Predicting personalized driver genes from the gene-patient associations through linear neighborhood propagation. The overview of our method is summarized in Fig. 1.

### Constructing personalized gene interaction network

Intrigued by LIONESS [29], for a given patient *p* and a group of patients as reference, such as the patients of a specific cancer data set in TCGA, we construct a personalized gene interaction network for the given patient* p* based on the statistical difference between the gene network built on all patients and the gene network built on all patients except the given patient* p*.

Pearson Correlation Coefficient (PCC) has been widely adopted to assess the correlations between the patients’ gene expression profiles to identify personalized driver genes. Following literature [19, 24], we first use all patients’ tumor gene expression data to compute the Pearson Correlation Coefficient (PCC) between the patients’ genes to evaluate the correlations between the patients’ gene expression profiles. Briefly, we first identified a group of tumor samples for the studied cancer type. The PCC of each pair of genes was calculated according to the expression data of the patient* p* to construct the gene interaction network *G*_*1*_. In the similar way, we use the tumor gene expression data of all patients excluding the patient* p* to compute the PCC between the patients’ genes and construct another gene interaction network *G*_*2*_. Then, all the edges with significantly differential correlations [23, 24] (i.e. *p* value < 0.05) were retained and used to construct the personalized gene interaction network for that cancer patient.

Finally, we remove the edges that exist in both *G*_*1*_ and* G*_*2*_, and remain the edges that only exist in *G*_*1*_ or else* G*_*2*_, and use the remaining edges of *G*_*1*_ and* G*_*2*_ to construct the personalized gene interaction network *G*_*p*_(*V*, *E*) for the patient *p*, where *V* and *E* are the node set and edge set of *G*_*p*_(*V*, *E*) respectively.

Note that, in the construction of *G*_*p*_(*V*, *E*) for the patient *p* of a cancer, remaining the edges, which only exist in *G*_*1*_ or else* G*_*2*_, between genes *i* and *j* is based on the observation: *G*_*1*_ is produced with the patient *p* and* G*_*2*_ is produced without the patient *p*, and the edge between genes *i* and *j* is only included in *G*_*1*_ or else* G*_*2*_, which implies the presence of this patient alters the association between genes *i* and *j* in *G*_*p*_(*V*, *E*), and therefore the interactions between genes *i* and *j* of this patient could have a relatively high correlation with this cancer. We thus remain these edges in *G*_*p*_(*V*, *E*).

On the other side, removing the edges, which exist in both of *G*_*1*_ and* G*_*2*_, between genes *i* and *j* is based on the observation: both of *G*_*1*_ and* G*_*2*_ include the edges between genes *i* and *j*, which implies the presence of this patient does not affect the association of genes *i* and *j* in *G*_*p*_(*V*, *E*), and therefore the interactions between genes *i* and *j* of this patient could have a relatively low correlation with this cancer. We thus remove these edges in *G*_*p*_(*V*, *E*).

In order to obtain accurate and reliable regulatory mechanism of personalized gene interaction network for each patient, based on the known gene interaction network retrieved from the existing gene interaction databases, we refine the personalized gene interaction network *G*_*p*_(*V*, *E*) by removing the edges(interactions) that are not supported by the known gene interaction network [30, 31]. The constructed personalized networks for different types of cancer are specific significantly as the constructed network are built upon the individual patients of a specific cancer dataset in TCGA. Meanwhile, the known gene interaction network data only serves to refine the constructed personalized interaction network. Note that the specific known gene interaction network can be specified by the users and the known gene interaction network ConsensusPathDB [30, 31] used in this work can be found at Additional file 1.

### Identifying potential gene-patient associations

After obtaining the personalized gene interaction network *G*_*p*_(*V*, *E*) for the patient *p*, we now try to determine potential gene-patient associations for the patient *p* based on* G*_*p*_(*V*, *E*). Note that, in *G*_*p*_(*V*, *E*), the interactions(edges) between genes *i* and *j* are possibly associated with current tumor for the patient *p*. A set of genes that are capable of interacting (connecting) with most genes of *G*_*p*_ could play central or driving role in controlling the gene interaction network and could most likely be potential driver genes for the patient *p* [2]. An intuitive way for discovering such potential genes is to find the genes that are able to maximumly cover the nodes of* G*_*p*_(*V*, *E*) [2, 24].

Based on this intuition, we adopt the following steps to identify potential driver genes for each patient in *G*_*p*_(*V*, *E*): (1) building a bipartite graph from the personalized gene interaction network *G*_*p*_(*V*, *E*), where the nodes of the left side are the nodes of *G*_*p*_(*V*, *E*) and the nodes of the right side are the edges of *G*_*p*_(*V*, *E*), and (2) determining the maximum matching set of the left side nodes to cover the right side nodes in the bipartite graph by using the well-known bipartite graph matching Hungarian algorithm [24, 32].

Specifically, for the personalized gene interaction network *G*_*p*_(*V*, *E*), we first transform *G*_*p*_(*V*, *E*) into a bipartite graph *G*(*L*,* R*, *E*_*B*_, *W*), where *L* = *V*, *R* = *E*, *E*_*B*_ is the edge set of the bipartite graph *G*(*L*,* R*, *E*_*B*_, *W*) and *W*_*v,u*_ $\in$ *W* is the weight of the edge (*v*,* u*)$\in$ *E*_*B*_,* v* $\in$ *L*,* u* $\in$ *R.* After building the bipartite graph *G*(*L*,* R*, *E*_*B*_, *W*), we try to find a maximum matching set *M* from *L*, which could maximumly cover the nodes of* R*, and choose the genes in the maximum matching set *M* as the potential driver genes for the patient *p*.

Next, we apply the well-known bipartite graph matching algorithm “Hungarian algorithm” [32] to find the maximum weighted bipartite matching in *G*(*L*,* R*, *E*_*B*_, *W*). In fact, the maximum weighted bipartite matching in *G*(*L*,* R*, *E*_*B*_, *W*) is an edge set *M* ⊆ *E*_*B*_ such that the sum of the edge weights ${\sum }_{(v,u)\in M}{W}_{v,u}$ is maximum and the nodes of each edge in *M* are different [33]. Following Hungarian algorithm, we can obtain the maximum matching set *M* from *L* by solving the following linear programming relaxation [32]:

1 $$\begin{aligned} max_{{x_{v,u} }} \mathop \sum \limits_{{\left( {v,u} \right) \in E_{B} }} & x_{v,u} W_{v,u} \\ s.t \mathop \sum \limits_{{u \in R:\left( {v,u} \right) \in E_{B} }} & x_{v,u} = 1, \quad \forall v \in L \\ & x_{v,u} \ge 0 \\ \end{aligned}$$

where the edge weight *W*_*v,u*_ of the bipartite graph is set to 1, *x*_*v,u*_ is an indicative variable, (*v*,* u*)$\in$ *E*_*B*_ is the edge between *v* $\in$ *L* and* u* $\in$ *R* in the bipartite graph *G*(*L*,* R*, *E*_*B*_, *W*). The solution of formula (1) is the maximum bipartite matching set *M* in *G*(*L*,* R*, *E*_*B*_, *W*).

Since the nodes(genes) in the maximum matching set *M* can maximumly cover the right side nodes of the bipartite graph *G*(*L*,* R*, *E*_*B*_, *W*) [33], these nodes(genes) could associate with most of the genes in *G*_*p*_(*V*, *E*) and play central or driving role in controlling the gene interaction network. Finally, we can choose the genes in the maximum matching set *M* as the potential driver genes for the patient *p*.

In the following, the potential driver genes of all patients in the tumor reference samples will be repeatedly produced in the similar way and the produced driver genes of all patients are used to construct the gene-patient association matrix *M*_*gp*_, where the rows are genes and the columns are patients in* M*_*gp*_. In matrix *M*_*gp*_, if gene *i* is a potential driver gene for patient *j* then *M*_*gp*_(*i*,*j*) = 1, otherwise *M*_*gp*_(*i*,*j*) = 0.

### Predicting driver genes

Previous studies indicated that the data point associations in matrix could be reconstructed and refined by using linear neighborhood similarity [34–36]. Intrigued by this, we utilize a linear neighborhood propagation model to refine the gene-patient associations in *M*_*gp*_ to detect personalized driver genes. In this model, we compute the linear neighborhood similarities of genes among patients in *M*_*gp*_, use a label propagation method based on the linear neighborhood similarities of genes to infer the unobserved gene-patient associations to refine *M*_*gp*_, and identify the personalized driver genes from the refined gene-patient associations.

Specifically, assume that there are *n* genes and *m* patients in *M*_*gp*_, we denote these *n* genes as feature vectors *y*_*i*_, *i* = 1, 2, …, *n* and consider these genes as data objects. This optimization problem can be formulated as the objective function:

2 $$\begin{aligned} & min_{{w_{{ii_{j} }} }} \left\| {y_{i} - \mathop \sum \limits_{{i_{j} :y_{{i_{j} }} \in N\left( {y_{i} } \right)}} w_{{ii_{j} }} y_{{i_{j} }} } \right\|^{2} = min_{{w_{{ii_{j} }} }} \mathop \sum \limits_{{i_{j} ,i_{k} :y_{{i_{j} }} ,y_{{i_{k} }} \in N\left( {y_{i} } \right)}} w_{{ii_{j} }} G_{{i_{j} ,i_{k} }}^{i} w_{{ii_{k} }} = w_{i}^{T} G^{i} w_{i} \\ & \quad \begin{array}{*{20}c} {{\text{s}}.{\text{t}}.} & {{ }\mathop \sum \limits_{{i_{j} }} w_{{ii_{j} }} = 1,} & {w_{{ii_{j} }} \ge 0.} \\ \end{array} \\ \end{aligned}$$

where *N*(*y*_*i*_) is a neighbor set of *y*_*i*_ with *k* (*k* = 1, 2, …, *n *− 1) nearest neighbors,* y*_*ij*_ denotes the *j*th neighbor of *y*_*i*_ and* w*_*iij*_ represents the contribution of* y*_*ij*_ to reconstruct *y*_*i*_, and* w*_*iij*_ can be regarded as the linear neighborhood similarity of *y*_*ij*_ and* y*_*i*_, *w*_*i*_ = (*w*_*ii1*_, *w*_*ii2*_, …, *w*_*iik*_)^*T*^. *G*^*i*^_*ij,ik*_ = (*y*_*i*_ − *y*_*ij*_)^*T*^(*y*_*i*_ − *y*_*ik*_) is the *j*th row and *k*th column of Gram matrix *G*^*i*^ [34–36].

In order to minimize the norm of reconstructive weight *w*_*i*_, the Tikhonov regularization term was added to prevent overfitting [34–36]. Then, we can rewrite the objective function as:

3 $$\begin{aligned} & min_{{w_{{ii_{j} }} }} \left\| {y_{i} - \mathop \sum \limits_{{i_{j} :y_{{i_{j} }} \in N\left( {y_{i} } \right)}} w_{{ii_{j} }} y_{{i_{j} }} } \right\|^{2} = w_{i}^{T} G^{i} w_{i} + \gamma w_{i}^{2} = w_{i}^{T} (G^{i} + \gamma I)w_{i} \\ & \quad \begin{array}{*{20}c} {{\text{s}}.{\text{t}}.{ }} & {\mathop \sum \limits_{{i_{j} }} w_{{ii_{j} }} = 1,} & {w_{{ii_{j} }} \ge 0.} \\ \end{array} \\ \end{aligned}$$

where *γ* is the regularization coefficient and is set to 1 for simplicity and *I* $\in$ *R*^*n*×*n*^ is identity matrix.

We first use standard quadratic programming [37] to solve (3), and the solutions* w*_*i*_ = (*w*_*ii1*_, *w*_*ii2*_, …, *w*_*iik*_)^*T*^ are the reconstructive weights of the data point *y*_*i*_, *i* = 1, 2, …, *n* and* k* = 1, 2, …, *n *− 1. Then we use these solutions to construct a weight matrix *S* $\in$ *R*^*n*×*n*^ and each entry of *S* can be regarded as the linear neighborhood similarity of genes. Based on the weight matrix *S*, we construct an undirected graph *G*_*dg*_ where the nodes are genes and the edge weights are the similarities of genes.

In the graph *G*_*dg*_, we utilize a label propagation method, which iteratively propagates the label information of driver genes on *G*_*dg*_, to discover the unobserved gene-patient associations to refine *M*_*gp*_. The initial associations of *n* genes and the patient *p*_*i*_ in* M*_*gp*_ can be regarded as the initial labels of *n* genes for the patient *p*_*i*_. In each propagation, every driver gene receives label information from the gene’s neighbors with proportion *α* and reserves the initial label with proportion 1 − *α*. The iteration is defined as:

4 $${F}_{i}^{t+1}=\alpha S{F}_{i}^{t}+(1-\alpha ){F}_{i}^{0}$$

where ${F}_{i}^{0}={({f}_{1i}^{0},{f}_{2i}^{0},\dots ,{f}_{ni}^{0})}^{T}$ represents the initial labels of *n* genes for the patient *p*_*i*_ and ${F}_{i}^{t}={({f}_{1i}^{t},{f}_{2i}^{t},\dots ,{f}_{ni}^{t})}^{T}$ denotes the predicted labels of *n* genes for the patient *p*_*i*_ at iteration *t* [34]. Considering all *m* patients, let ${F}^{t}=({F}_{1}^{t},{F}_{2}^{t},\dots ,{F}_{m}^{t})$, and the iteration process can be formulated in matrix form as follows:

5 $${F}^{t+1}=\alpha S{F}^{t}+(1-\alpha ){F}^{0}$$

We can use Eq. (5) to update the label matrix. Finally, Eq. (5) will be converged to the following:

6 $${F}^{*}=(1-\alpha ){(I-\alpha S)}^{-1}{F}^{0}$$

F^*^ is the predicted gene-patient association score matrix. Then the gene-patient associations in *M*_*gp*_ are refined to F^*^ by inferring the unobserved gene-patient associations through propagating the label information of driver genes. We can obtain the prediction scores of the genes for each patient from F^*^ and identify the highly rank genes as the personalized driver genes of each patient for further analysis. More details on the convergence inference of the label propagation can be found in literature [35].

## Results

### Performance comparison

In this section, we validate the effectiveness of LPDriver by comparing it with other ten state-of-the-art methods including five personalized cancer driver identification methods including PNC [24], WMDS.netP [23], DawnRank [18], SCS [19] and SSN [38], and five population level cancer driver identification methods including DriverNet [12], OncoDriveFM [8], MutSigCV [7], DriverML [9] and ActiveDriver [10].

Based on the data availability of the compared methods, we used twelve TCGA [26] cancer datasets as the test datasets: Breast invasive carcinoma (BRCA), Colon adenocarcinoma (COAD), Head and neck squamous cell carcinoma (HNSC), Kidney chromophobe (KICH), Kidney renal clear cell carcinoma (KIRC), Kidney renal papillary cell carcinoma (KIRP), Liver hepatocellular carcinoma (LIHC), Lung adenocarcinoma (LUAD), Lung squamous cellcarcinoma (LUSC), Prostate adenocarcinoma (PRAD), Papillary thyroid carcinoma (THCA) and Uterine corpus endometrial carcinoma (UCEC).

The known driver genes of Cancer Gene Census (CGC v.84) [28] and the Network of Cancer Genes (NCG 6.0) [27] database are used as the ground truth for assessing predicted driver genes. In cancer research, CGC and NCG are commonly used cancer gene datasets for validating driver genes predicted by computational methods. In total, 711 known cancer genes and 616 cancer census genes are downloaded from CGC and NCG gene lists (see Additional file 2).

The predicted driver genes annotated in the NCG and CGC were utilized to compute the F-measure to evaluate the performance of different methods [2, 24]. F-measure is computed by the following equation:

7 $$F-measure=\frac{2*precision*recall}{precision+recall}$$

where the precision denotes the ratio of correctly identified driver genes to all identified driver genes and the recall denotes the ratio of correctly identified driver genes to the driver genes of NCG and CGC [2, 24]. In the performance comparisons, LPDriver finds potential driver genes from 12 cancer datasets with the proportion parameter *α* = 0.5 and choose the identified genes appearing among over 80% patients in each dataset as the resulting driver genes. For these ten comparative methods, we obtained their identified driver genes for twelve TCGA cancer datasets from the WMDS.netP paper [23]. These identified driver genes of the comparison methods were obtained by using the same TCGA cancer type datasets based on the default parameter values provided in their papers.

**Fig. 2:**
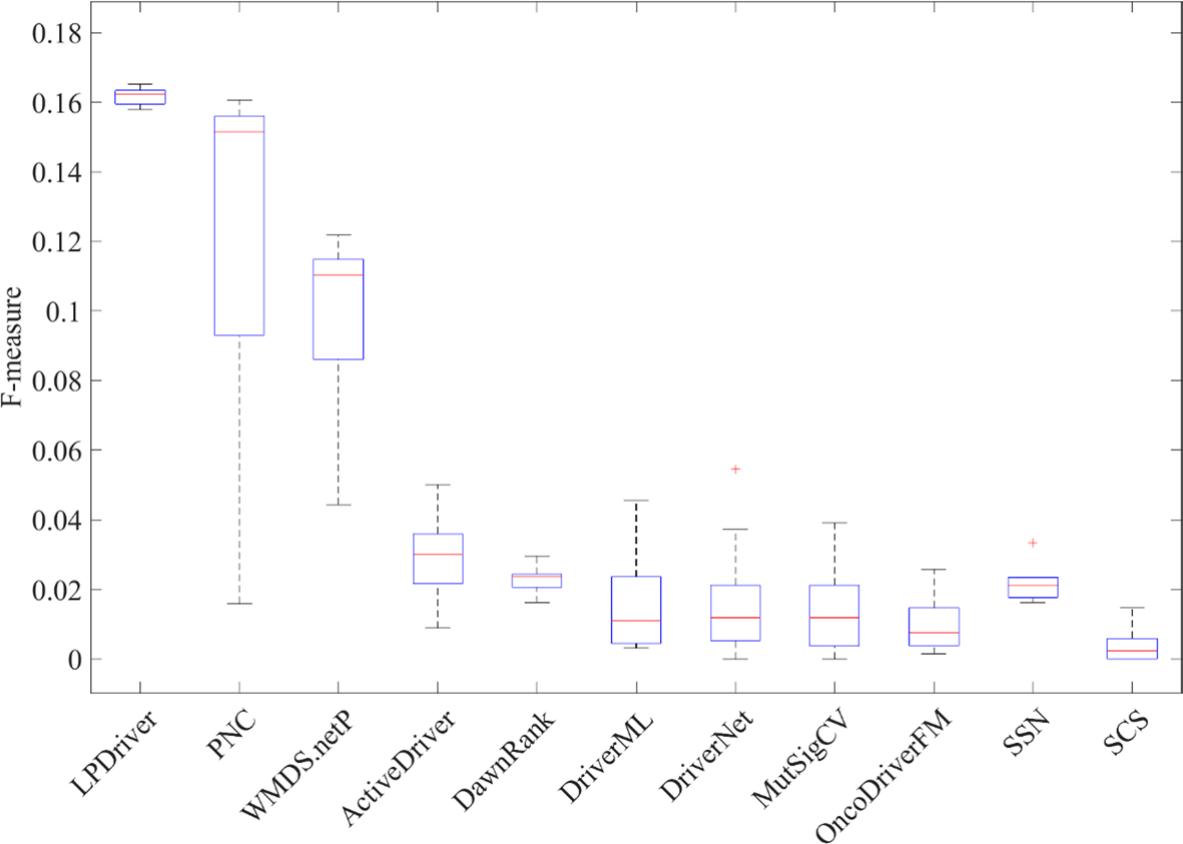
Significant enrichment F-measures of the results from 11 methods. For each method, the F-measure values are the average results of twelve TCGA cancer datasets including BRCA, COAD, HNSC, KICH, KIRC, KIRP, LIHC, LUAD, LUSC, PRAD, THCA and UCEC

Figure 2 shows the F-measures of the predicted cancer driver genes from different methods. As can be seen in Fig. 2, we can find that LPDriver outperforms other comparative methods in terms of the average values of F-measure. This result indicates that LPDriver is an effective method for predicting cancer driver genes.

**Fig. 3:**
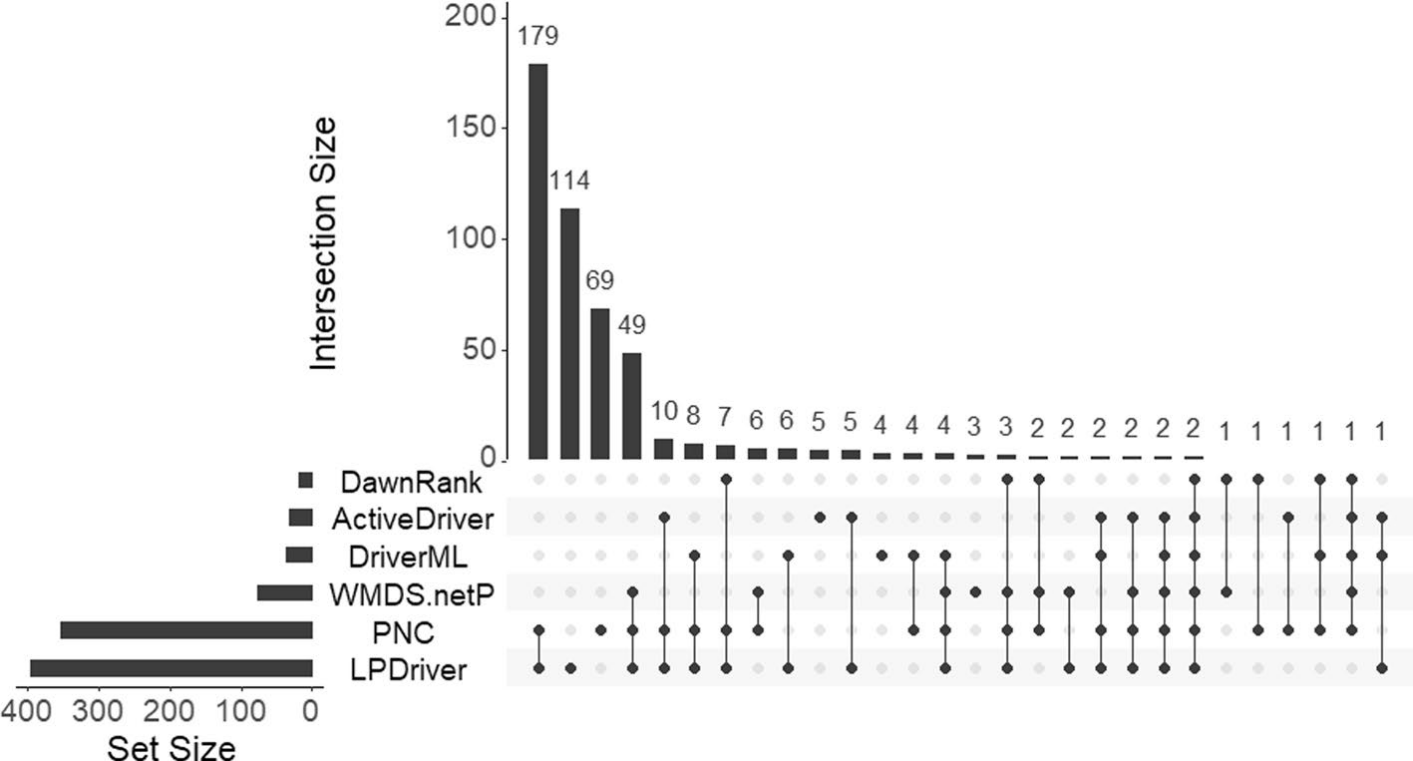
Overlap among different methods for BRCA. The chart depicts the overlap between the driver genes detected by the six methods (LPDriver, PNC, WMDS.netP, DawnRank, ActiveDriver and DriverML) for BRCA. The horizontal bars situated at the bottom left signify the number of the identified driver genes that have been validated in both NCG and CGC. Meanwhile, the vertical bars, in conjunction with the dotted lines, collectively depict the overlaps among the validated driver genes of different methods

Moreover, to verify whether LPDriver detects similar driver genes as other top 5 performing methods such as PNC, WMDS.netP, DawnRank, ActiveDriver and DriverML, we also compare the overlap between the prediction results of these methods. The discovered cancer drivers of these comparison methods are validated with both NCG and CGC and intersected to find the overlaps. Figure 3 shows the overlap among different methods for BRCA. The prediction overlap of the remaining eleven datasets can be found in the Additional file 3. As we can see in Fig. 3, although different methods have identified similar driver genes, the prediction overlap between LPDriver and other methods demonstrate that LPDriver is able to identify cancer driver genes that have not yet been identified by other methods. The complementarity of these methods can be utilized to maximize the prediction accuracy of cancer driver genes.

### Effect of proportion parameter α

LPDriver relies on propagating label information of genes in the graph *G*_*dg*_ to mine gene-patient associations to detect personalized driver genes. The parameter *α* is used to adjust the gene label propagation proportion for each gene’s initial label and the label information from neighborhood genes, which is critical in propagating gene labels.

In order to learn the impact of *α* on the performance of LPDriver, we calculate the F-measures of LPDriver with different *α* on twelve cancer datasets and the results are shown in Table 1. As we can see in Table 1, for 7 out of 12 datasets (i.e. COAD, HNSC, KIRC, LUAD, LUSC, PRAD and THCA), LPDriver receives the best F-measure in the case of *α* = 0.5. Meanwhile, LPDriver yields the best F-measure for BRCA and LIHC when *α* = 0.4, and gives the best F-measure for KICH and UCEC when *α* = 0.6. This result indicates that the gene’s initial label and the label information from neighborhood genes contribute almost equally to the identification of cancer driver genes. We thus identically set *α* as 0.5.

**Table 1:** Performance comparison with different *α* on twelve cancer datasets

*α*	BRCA	COAD	HNSC	KICH	KIRC	KIRP	LIHC	LUAD	LUSC	PRAD	THCA	UCEC
0.1	0.1501	0.1537	0.1521	0.1426	0.1465	0.1446	0.1564	0.1589	0.1564	0.1489	0.1496	0.1464
0.2	0.1572	0.1553	0.1613	0.1413	0.1489	0.1475	0.1588	0.1565	0.1585	0.1523	0.1534	0.1423
0.3	0.1630	0.1573	0.1565	0.1535	0.1534	0.1496	0.1613	0.1603	0.1605	0.1589	0.1610	0.1520
0.4	**0.1665**	0.1616	0.1593	0.1598	0.1580	0.1553	**0.1623**	0.1591	0.1626	0.1623	0.1586	0.1480
0.5	0.1619	**0.1631**	**0.1624**	0.1578	**0.1651**	0.1582	0.1605	**0.1615**	**0.1626**	**0.1648**	**0.1635**	0.1584
0.6	0.1617	0.1605	0.1612	**0.1612**	0.1621	0.1610	0.1605	0.1603	0.1579	0.1617	0.1586	**0.1612**
0.7	0.1577	0.1586	0.1583	0.1576	0.1634	**0.1634**	0.1574	0.1584	0.1586	0.1638	0.1565	0.1576
0.8	0.1536	0.1592	0.1546	0.1562	0.1602	0.1621	0.1532	0.1565	0.1543	0.1623	0.1555	0.1563
0.9	0.1488	0.1571	0.1525	0.1553	0.1580	0.1582	0.1517	0.1543	0.1486	0.1589	0.1584	0.1534

*Significant of bold values demonstrate the best F-measure of LPDriver with different *α* on each dataset

### Influence of different reference networks

In order to comprehensively learn the influence of the reference gene interaction network on the network-based methods, we also evaluated the performance of LPDriver and other top 2 performing network-based methods PNC [24] and WMDS.netP [23] using different reference networks. These reference gene interaction networks are ConsensusPathDB [30, 31], HumanNet [39], StringNet [40] and the best performing reference network used in PNC, which is marked as network 6 in literature [24] and is called PNCNet in this work. Table 2 summarizes the number of genes and interactions from each of these four networks. (See Additional file 1 for more details of these networks).

**Table 2:** The gene number (Nodes) and interaction number (Edges) in four networks ConsensusPathDB, PNCNet, HumanNet and StringNet

	ConsensusPathDB	PNCNet	HumanNet	StringNet
Nodes	4912	9160	15,351	11,302
Edges	96,256	104,153	158,499	273,210

**Fig. 4:**
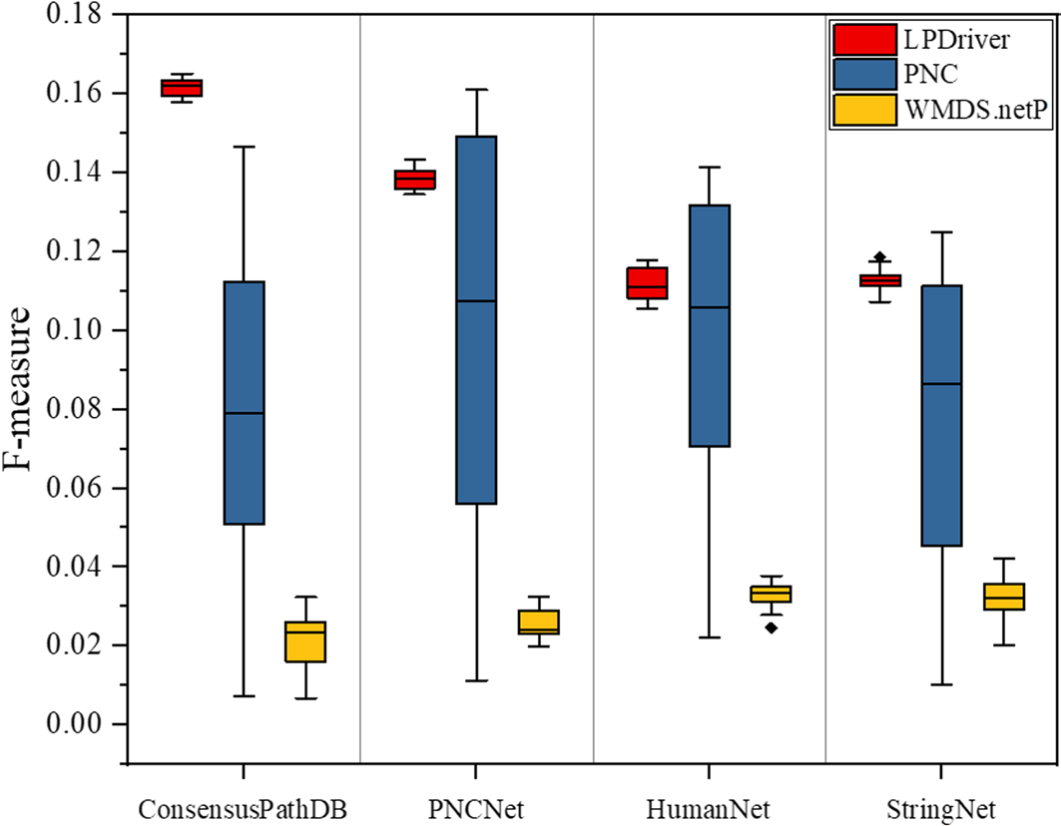
Average F-measures of LPDriver, PNC and WMDS.netP on twelve cancer data sets using different reference gene interaction networks (i.e. ConsensusPathDB, PNCNet, HumanNet and StringNet)

To obtain fair and convincing comparison results, we obtained the source codes of PNC and WMDS.netP from their literatures and ran these two comparative algorithms according to the default values suggested by their papers. Specifically, PNC sets to its default *p* value threshold 0.05 and WMDS.netP sets to its default hyperparameter *γ* 0.01. In the comparison, all compared algorithms were run on a computer with an Intel Xeon 6130 and 208GB RAM. The running operating system is Linux. We then compared the performance of LPDriver, PNC and WMDS.netP using four reference gene interaction networks on 12 cancer datasets and the comparison results are shown in Fig. 4.

As shown in Fig. 4, for using different reference gene interaction networks, LPDriver achieves better performance than PNC and WMDS.netP. This indicates that LPDriver is an effective network-based method for predicting cancer driver genes using different reference networks. Moreover, as we can see in Fig. 4, the F-measures of these three methods vary for different reference networks. This demonstrates that reference network has direct impact on these four network-based methods in deed. Interestingly, in Fig. 4, we can observe that, the variances of the F-measures for PNC and LPDriver using different reference networks are larger than those of WMDS.netP. These results reveal that PNC and LPDriver are more sensitive to reference network than WMDS.netP, and the use of proper reference network may enable LPDriver and PNC to obtain better performance.

### Ablation study

In this section, we will evaluate the effectiveness of different parts of LPDriver by ablation study. In the second step, LPDriver identifies the potential driver genes by finding the genes in the maximum matching set of gene-interaction bipartite graph and we call this step as BGGM for simplicity. In order to evaluate the effectiveness of the step BGGM, we replace the step BGGM of LPDriver with three classical network control methods including NCUA [24], MMS [24, 25] and MDS [24, 25] respectively to find driver genes in the gene-interaction bipartite graph. We constructed three modes for LPDriver, namely LPDriver_NCUA, LPDriver_MMS and LPDriver_MDS. Specifically, LPDriver_NCUA denotes the step BGGM of LPDriver is replaced by NCUA. LPDriver_MMS denotes the step BGGM of LPDriver is replaced by MMS. LPDriver_MDS denotes the step BGGM of LPDriver is replaced by MDS.

**Fig. 5:**
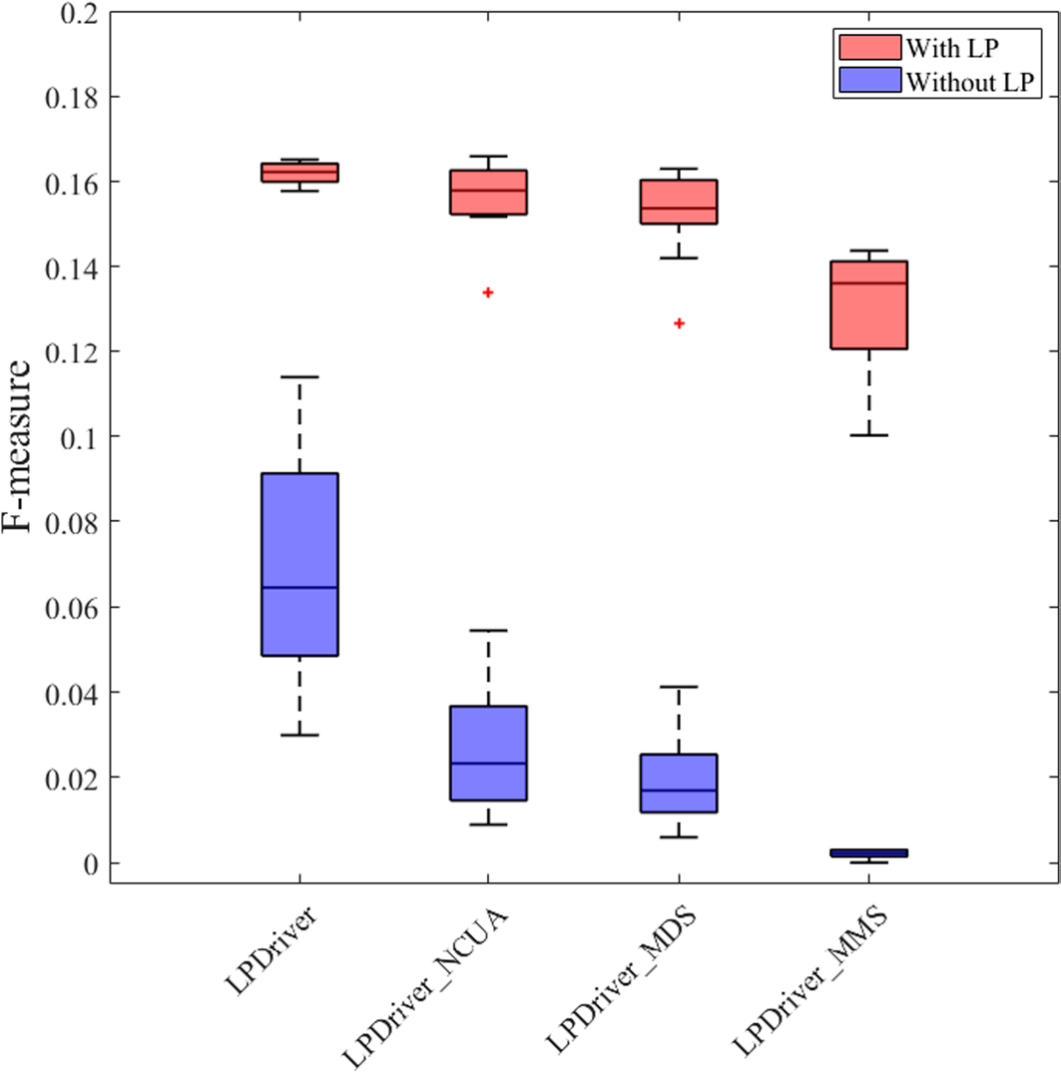
F-measures of LPDriver, LPDriver_NCUA, LPDriver_MMS and LPDriver_MDS. “With LP” and “Without LP” denote that the LPDriver’s modes are performed with and without using linear neighborhood propagation respectively. The x-axis shows four LPDriver’s modes and the y-axis is for F-measure. For each mode, the F-measure value is the result of twelve cancer datasets including BRCA, COAD, HNSC, KICH, KIRC, KIRP, LIHC, LUAD, LUSC, PRAD, THCA and UCEC

Figure 5 shows the F-measures of the predicted cancer driver genes of four different LPDriver’s modes on twelve cancer datasets. Furthermore, in Fig. 5, to estimate the effect of predicting driver genes using linear neighborhood propagation in LPDriver, we performed these four LPDriver’s modes with and without using linear neighborhood propagation respectively.

From Fig. 5, we can see that, for the mode performed with using linear neighborhood propagation, the F-measure of LPDriver is better than that of LPDriver_NCUA, LPDriver_MMS and LPDriver_MDS. Similarly, in Fig. 5, for the mode performed without using linear neighborhood propagation, the F-measure of LPDriver is higher than that of LPDriver_NCUA, LPDriver_MMS and LPDriver_MDS as well. These results demonstrate that finding genes in the maximum matching set of the gene-interaction bipartite graph could be an effective way of identifying personalized driver genes for LPDriver.

Moreover, as can be seen in Fig. 5, the F-measures of the LPDriver’s modes performed with using linear neighborhood propagation are much better than those of the LPDriver’s modes performed without using linear neighborhood propagation. These results illustrate the effectiveness of predicting driver genes via linear neighborhood propagation in LPDriver.

### Discovering personalized driver genes

The personalized driver genes may vary for different patients due to cancer heterogeneity [3]. Hence, the genes that rarely mutated in a population could potentially drive the cancer development of each patient, and these genes are also called rare driver genes [3]. The rare driver genes are likely ignored by mutation frequency-based methods [3]. In this section, we discuss the rare driver genes predicted by LPDriver to learn the effectiveness of detecting personalized driver genes for LPDriver at the individual level.

**Fig. 6:**
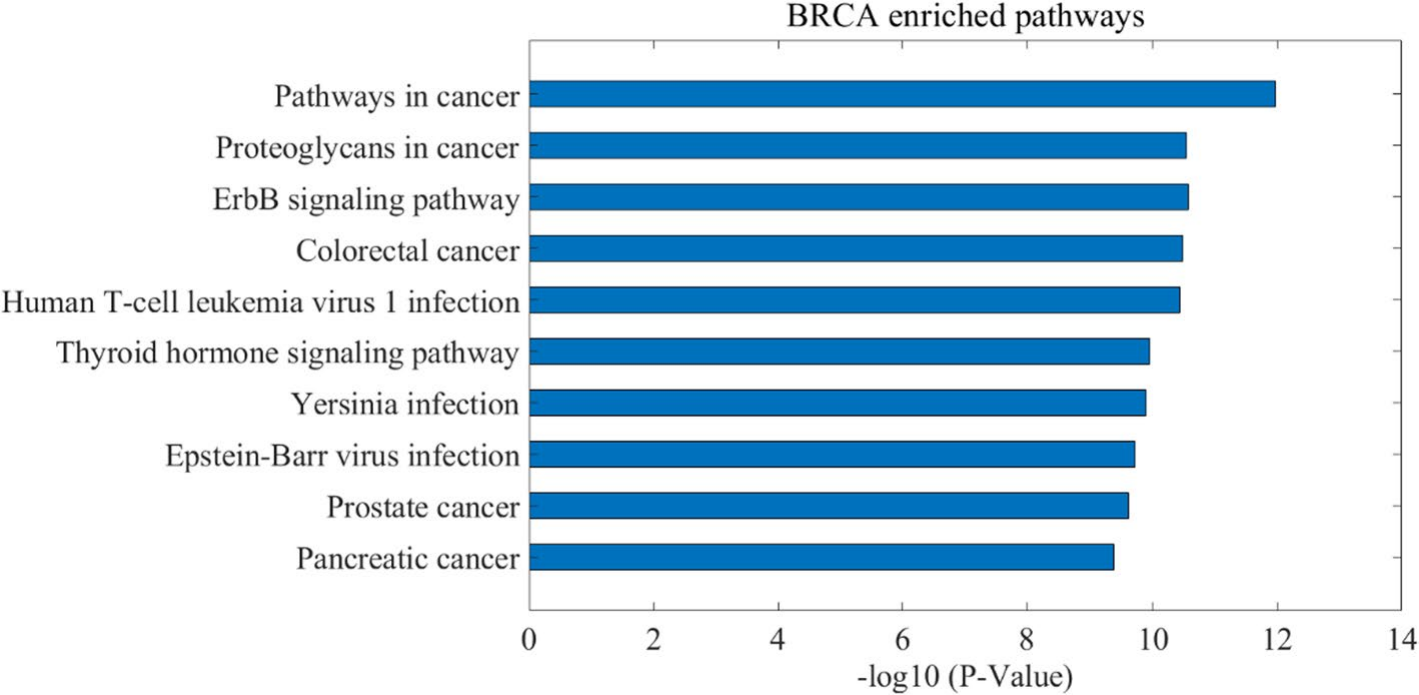
Top 10 enriched KEGG pathways of the personalized rare driver genes on BRCA cancer dataset. The Y-axis indicates the name of enriched KEGG pathway. The X-axis represents the opposite value of log transformed *p* value. The larger value of X-axis indicates that the genes are more significantly enriched in the pathway

Here, we define the genes that are mutated in no more than 5% of patients in a cancer dataset and is ranked top 100 of a patient’s potential driver genes as the personalized rare driver genes. We used DAVID [41–43] tools to perform the functional enrichment analysis on these personalized rare driver genes identified by LPDriver against the pathway database Kyoto Encyclopedia of Genes and Genomes (KEGG) [44]. Taking BRCA as an example, the top 10 enriched KEGG pathways of the personalized rare driver genes are shown in Fig. 6. The identified rare driver genes and the top 10 enriched pathways of these driver genes for BRCA, COAD, HNSC, KICH, KIRC, KIRP, LIHC, LUAD, LUSC, PRAD, THCA and UCEC cancers are given in the Additional file 4 and file 5 respectively. From these results, we can find that these rare driver genes are enriched in some critical pathways related with cancer [41]. It is noted that, in the functional enrichment analysis [2, 3, 24, 45], the higher -log(*p* value) value of the pathway is, the better enriched significance of the pathway is. Notably, we chose three rare driver genes *GSK3B*, *SP1* and *XRCC6*, which have the minimum occurrence frequency as mutated genes in BRCA cancer dataset, to analyze.

Taking the gene *GSK3B* as example, it ranks the top 2.3% of the potential driver genes for the patient TCGA-BH-A0DL and ranks the top 2.8% of the potential driver genes for the patient TCGA-BH-A18M. Even though the mutation frequency of gene *GSK3B* in BRCA cancer dataset is only 0.504%, our LPDriver still ranks *GSK3B* in top 10% of all personalized driver genes with mutation in BRCA cancer dataset. Additionally, it was reported that the overexpression of *GSK3B* promotes the development of multiple cancers [46]. Similar results can also be observed in the patients with genes *SP1* and *XRCC6*. *SP1* plays a critical role in the development of pancreatic cancer [47] and *XRCC6* is a risk allele for breast cancer [48]. These two rare mutation genes *SP1* and *XRCC6* are also obviously ranked ahead in the personalized driver genes for other patients in BRCA cancer dataset. Even when some driver genes rarely mutate in a cohort, LPDriver still uncovers and promotes these genes for personalized therapies. In short, the above results demonstrate that LPDriver is able to detect personalized driver genes by their network characteristics even if the mutation profiles of the genes are hidden.

### Statistic analysis of the identified driver genes

**Fig. 7:**
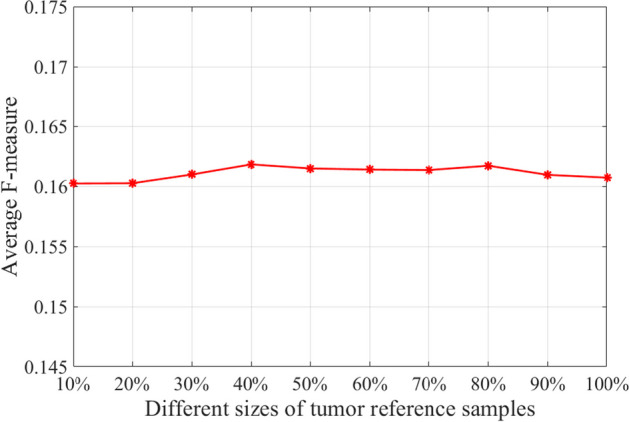
Average F-measures of LPDriver for different sizes of tumor reference samples. The y-axis indicates the average F-measures across the twelve datasets (i.e. BRCA, COAD, HNSC, KICH, KIRC, KIRP, LIHC, LUAD, LUSC, PRAD, THCA and UCEC). The x-axis shows the size of tumor reference samples used to construct gene interaction network, e.g. 10% indicates 10% of the tumor reference samples in the cancer data set are used to construct gene interaction network

LPDriver constructs personalized gene interaction network by using a group of tumor reference samples and the size of tumor reference samples may influence the effect of identifying driver genes. In order to learn the impact of tumor reference sample size to the performance of LPDriver, we apply LPDriver to identify driver genes on twelve cancer datasets (i.e. BRCA, COAD, HNSC, KICH, KIRC, KIRP, LIHC, LUAD, LUSC, PRAD, THCA and UCEC) by using different sizes of tumor reference samples. The average F-measures of LPDriver for each size of tumor reference samples across the twelve datasets are shown in Fig. 7. As can be seen in Fig. 7, the F-measure values keep stable with different sizes of tumor reference samples. This result indicates that the performance of LPDriver is not greatly affected by the size of tumor reference samples to some extent.

### Detecting potentially novel driver genes

In order to assess the ability of LPDriver on detecting potentially novel driver genes, we first used LPDriver to search for the top-ranked 100 driver genes from the breast cancer dataset BRCA while not in NCG and CGC, and obtained 42 potentially novel driver genes (see Additional file 6). We then used DAVID [41] tools to perform functional enrichment analysis on these 42 obtained driver genes against Genetic Association Database (GAD) which records the genes associated with diseases [49]. Interestingly, 36(85.7%) of these 42 genes are involved in GAD, and 28 (66.7%) genes are related with cancer, 18 genes are enriched for “Breast Cancer” (*p* value = 9.3 × 10^–4^, FDR = 6.6 × 10^–2^). Particularly, it has been confirmed that *ACTB* (actin beta) (ranked the 3th in patient TCGA-E2-A158 and the 5th in patient TCGA-BH-A1EU), is distinctly associated with the metastatic ability of human colon adenocarcinoma cells [50] and accumulating evidence demonstrates that *ACTB* is irregularly expressed in a variety of cancers and affects the metastasis and invasiveness of tumors [51].

Moreover, we performed enrichment analysis on these 42 genes against three pathway databases Kyoto Encyclopedia of Genes and Genomes (KEGG) [52], Gene Ontology (GO) [53, 54] and Reactome [55], the results show that these 42 genes are enriched for “Viral carcinogenesis” (KEGG pathway, *p* value = 4.8 × 10^–11^, FDR = 6.3 × 10^–9^), “R-HSA-2894862” (Reactome pathway, *p* value = 2.3 × 10^–9^, FDR = 2.5 × 10^–7^), and “viral process” (GO biological process, *p* value = 3.4 × 10^–8^, FDR = 2.1 × 10^–5^). Specifically, *RBPJ* (ranked 8th in patient TCGA-BH-A0BZ and 9th in patient TCGA-BH-A0BJ) was reported as a potential oncogene in certain cancers and the upregulation of *RBPJ* can induce pancreatic cancer [56], although *RBPJ* has not been listed in NGC and CGC under current version yet.

Driver genes may play different roles in cancer. Based on the identified driver genes from BRCA, we accordingly categorized some of these potentially novel driver genes that were recently reported to associate with cancer into four types based on their roles in the development of cancer [1]. The four types are the direct driver gene, induced driver gene, target driver gene and biomarker driver gene. These categorized genes are summarized in Additional file 7. In the following we discuss some of these categorized genes to learn the effectiveness of identifying potentially novel driver genes for LPDriver. The direct driver gene is the driver gene that was reported to directly cause the cancer [57]. For example, *CDK2*(ranked 16th in patient TCGA-BH-A18M) was recently discovered to be essential for the proliferation of prostate cancer cell [58]. Moreover, accumulating evidence indicates that *MED23* (ranked 9th in patient TCGA-E2-A1LH) plays an oncogenic role in the development of NSCLC (a non-small cell lung cancer) and influences the invasiveness and development of tumors [59].

The induced driver gene can exert its action on other genes or proteins to cause cancer [60]. For instance, *PCAF *(ranked 11th in patient TCGA-E9-A1RF), is recently reported to have positive role for inducing the acetylation of Glycerol 3-phosphate dehydrogenase (an enzyme in glycolysis) to promote cell proliferation in liver tumor [61]. Besides, due to the decrement of expression level, the attenuated function of gene *SIN3A* (ranked 93th in patient TCGA-E9-A1N6) may lead to the epigenetic deregulation of the growth-associated genes, which results in the oncogenesis of lung cancer cells [62]. Basically, the direct and induced driver genes predicted by LPDriver could help us to study the cause of cancer on genomic level.

On the other hand, the target driver gene could serve as the therapy target for curing cancer [63]. For example, recently, the overexpression of *SKIP* (ranked 10th in patient TCGA-BH-A1FD) was included in the pathogenesis and diagnosis of breast cancer, which could possibly serve as a future therapeutic target for breast cancer [64]. Besides, *SH3KBP1*(ranked 10th in patient TCGA-BH-A1FD) was reported to serve as a new regulator of carcinogenic EGFR (Epidermal Growth Factor Receptor), and it could also serve as a potential therapy target for GBM (Glioblastoma multiforme, a kind of cancer) patients with EGFR activation [65].

The biomarker driver gene could serve as biomarker for detecting the existence of cancer cell [66]. For example, a recent report showed that *RPA1* (ranked 8th in patient TCGA-E2-A15M) works as a presumed oncogene in tumorigenesis and serves as a prognosticative biomarker for colorectal cancer [67]. Also, the upregulation of *HnRNPM* (ranked 13th in patient TCGA-BH-A0H9) is contained in the human colorectal epithelial tumorigenesis and could serve as a tumor biomarker for colorectal cancer. These biomarker and target driver genes detected by LPDriver could help people to find the existence of cancer cell and provide potential therapy target for curing cancer.

In summary, the abovementioned results demonstrate that LPDriver is also an effective method for detecting potentially novel cancer driver genes.

## Discussion and conclusion

In this work, we propose LPDriver, a novel computational method for predicting personalized cancer driver genes. LPDriver offers several distinct advantages over its counterparts. First, LPDriver innovatively explores gene-patient associations within personalized gene networks to uncover functionally similar genes among patients. A key differentiator in this step is that LPDriver does not rely on known driver genes or mutation data to detect driver genes. This novelty accelerates and simplifies the identification process of cancer driver genes. Meanwhile, unlike other network-based methods, LPDriver identifies potential driver genes by selecting genes from the maximum matching set of personalized gene networks. This maximizes the coverage of gene network edges while preserving room for further exploration of driver genes, and strikes a balance between comprehensiveness and specificity in identifying candidate driver genes.

We have conducted comprehensive experiments on multiple cancer datasets from TCGA, benchmarking LPDriver against the state-of-the-art methods. LPDriver's superior performance in the experimental comparison demonstrates its effectiveness in detecting cancer driver genes. Notably, LPDriver excels in identifying known cancer driver genes, while also revealing potentially novel driver genes that are documented to be cancer-related. LPDriver thus can serve as an effective and valuable complement to the existing toolkit for identifying cancer driver gene, ultimately contributing to a comprehensive understanding of cancer genetics.

Despite the effectiveness of LPDriver in identifying cancer driver genes, some limitations remain. LPDriver constructs personalized gene networks upon the same known gene interaction networks and the gene expression data of a specific cancer. The information of specific cancer could be lost in the construction of personalized gene networks. A further extension is to utilize the gene interactions that are specific to a cancer under consideration to initiate the construction of personalized gene networks for a specific cancer. Moreover, as a future work, a variety of the biological features of genes and cancers, such as sequence profiles of genes or miRNA, could be incorporated to further promote the identification performance. Furthermore, for simplicity, LPDriver sets the edge weight of the bipartite graph of gene network to 1 to find the maximum matching in the bipartite graph for inferring potential gene-patient associations. In the future, more gene interaction information could be integrated to enrich the edge weight of the bipartite graph of gene network, which may help to find more accurate gene-patient associations.

### Supplementary Information

**Additional file 1**: The gene interaction network.

**Additional file 2**: The list of Cancer Genes Census (CGC) and Network of Cancer Genes (NCG).

**Additional file 3**: The prediction overlap of the remaining 11 datasets.

**Additional file 4**: The identified rare driver genes.

**Additional file 5**: The top 10 enriched pathways of the identified rare driver genes.

**Additional file 6**: The 42 potentially novel driver genes.

**Additional file 7**: The categorized genes identified by LPDriver.

## Data Availability

The source code and data are available at https://github.com/hyr0771/LPDriver.

## Abbreviations

TCGA: The cancer genome atlas
CGC: Cancer gene census
NCG: Network of Cancer Genes
LP: Linear neighborhood propagation
PCC: Pearson correlation coefficient
BRCA: Breast invasive carcinoma
COAD: Colon adenocarcinoma
HNSC: Head and neck squamous cell carcinoma
KICH: Kidney chromophobe
KIRC: Kidney renal clear cell carcinoma
KIRP: Kidney renal papillary cell carcinoma
LIHC: Liver hepatocellular carcinoma
LUAD: Lung adenocarcinoma
LUSC: Lung squamous cellcarcinoma
PRAD: Prostate adenocarcinoma
THCA: Papillary thyroid carcinoma
UCEC: Uterine corpus endometrial carcinoma
KEGG: Kyoto Encyclopedia of Genes and Genomes
GO: Gene ontology
