# Supplementary material for: Exploring gene-patient association to identify personalized cancer driver genes by linear neighborhood propagation
Source: BMC Bioinformatics. 2024 Jan 22;25:34. doi: 10.1186/s12859-024-05662-4 (PMC10804660; doi:10.1186/s12859-024-05662-4)
Supplement: Supplementary file 3 — Additional file 3: The prediction overlap of the remaining 11 datasets. [file 12859_2024_5662_MOESM3_ESM.docx]

#### Additional file 3. The prediction overlap of the remaining 11 datasets.


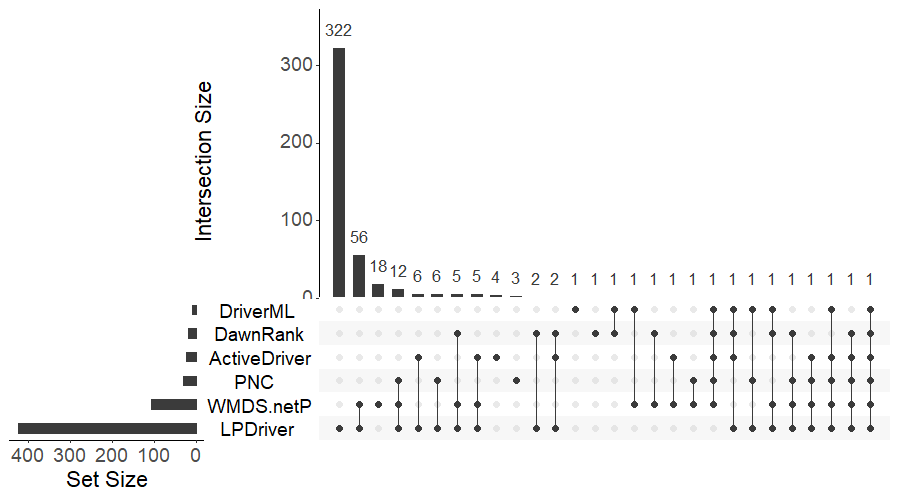


Fig 1. Overlap among different methods for COAD.


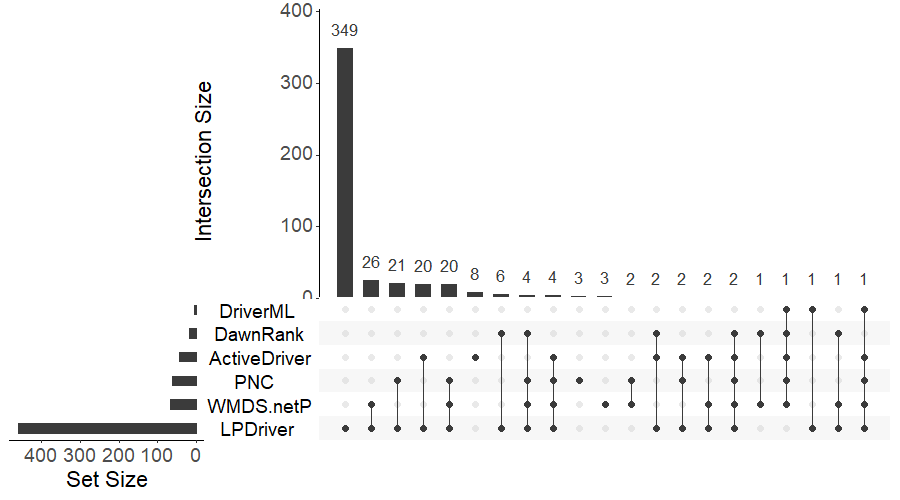


Fig 2. Overlap among different methods for HNSC.


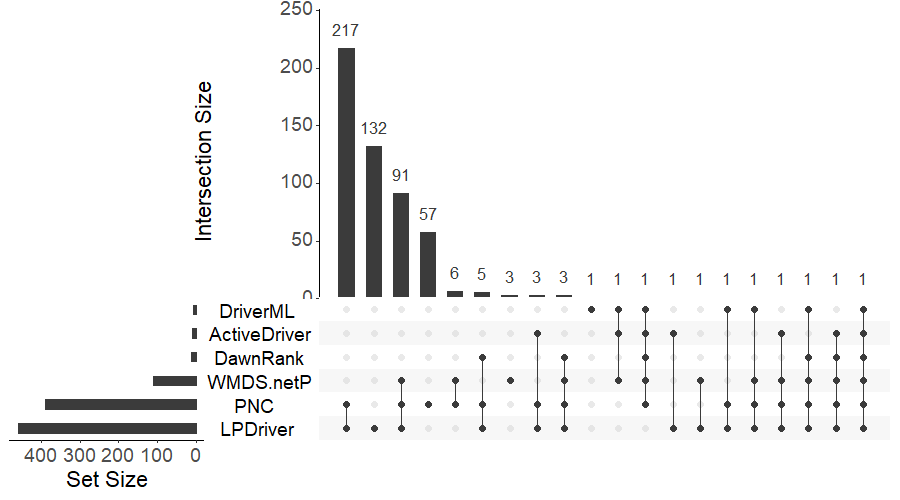


Fig 3. Overlap among different methods for KICH.


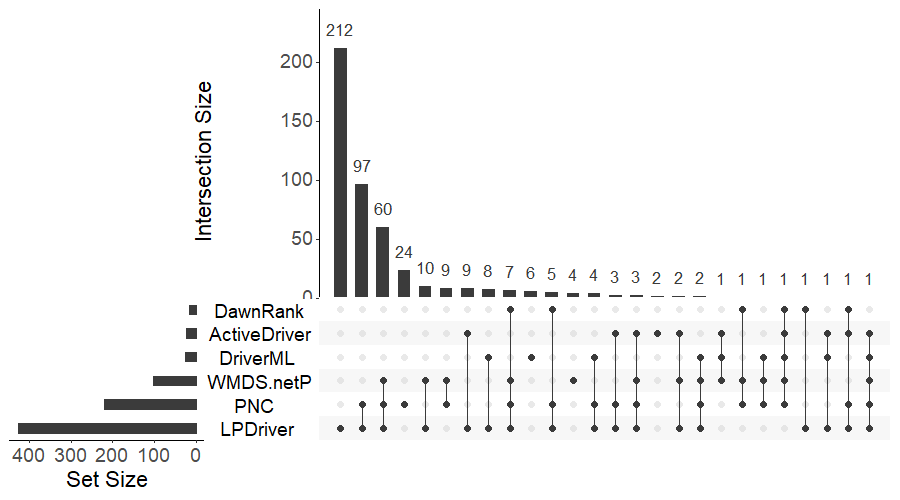


Fig 4. Overlap among different methods for KIRC.


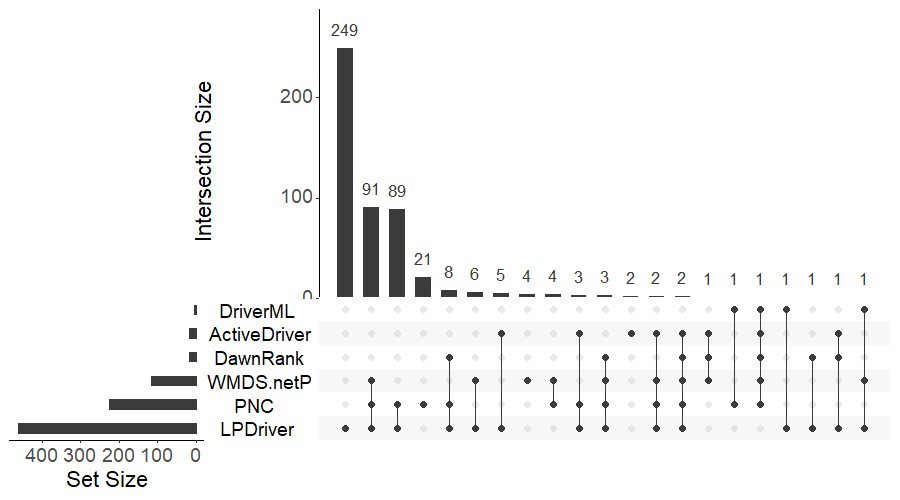


Fig 5. Overlap among different methods for KIRP.


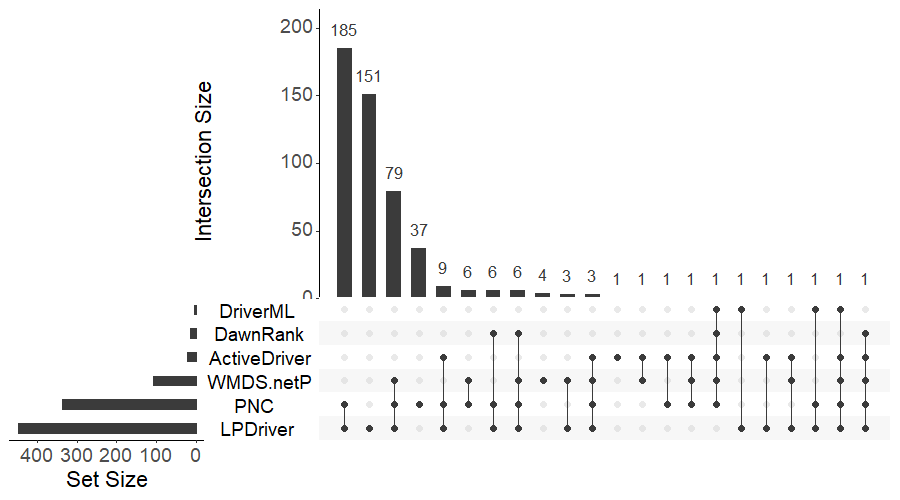


Fig 6. Overlap among different methods for LIHC.


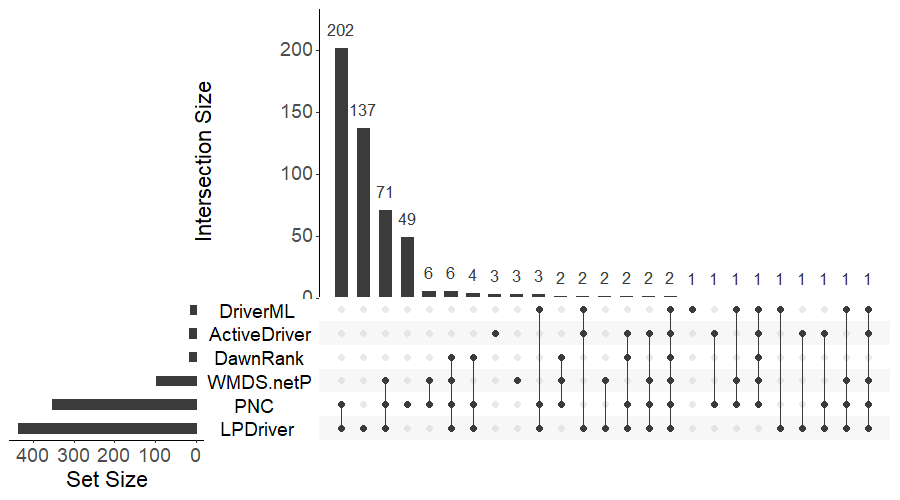


Fig 7. Overlap among different methods for LUAD.


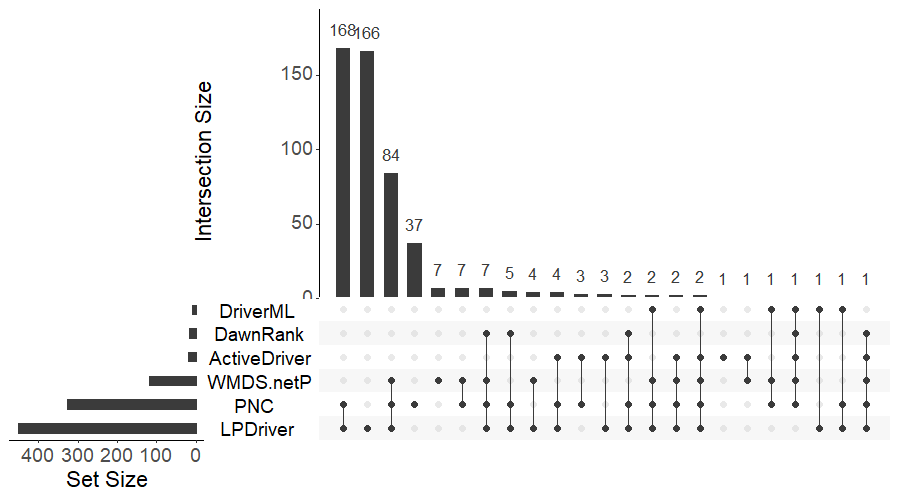


Fig 8. Overlap among different methods for LUSC.


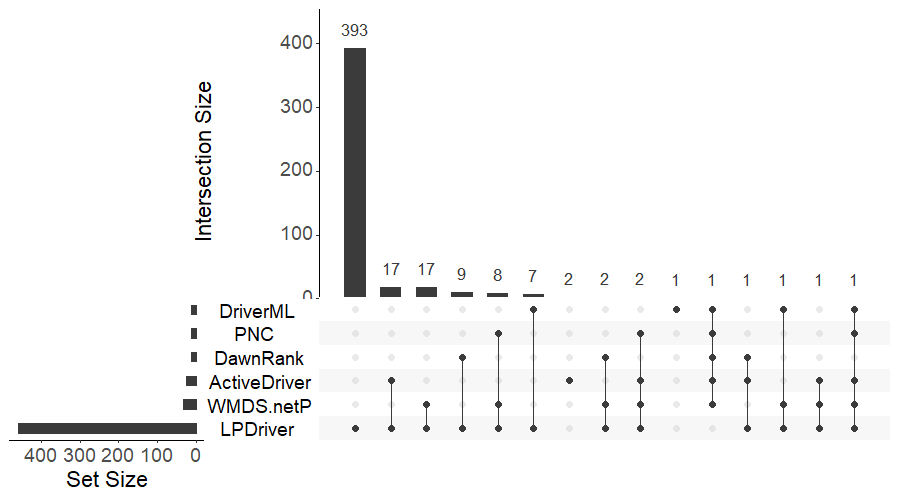


Fig 9. Overlap among different methods for PRAD.


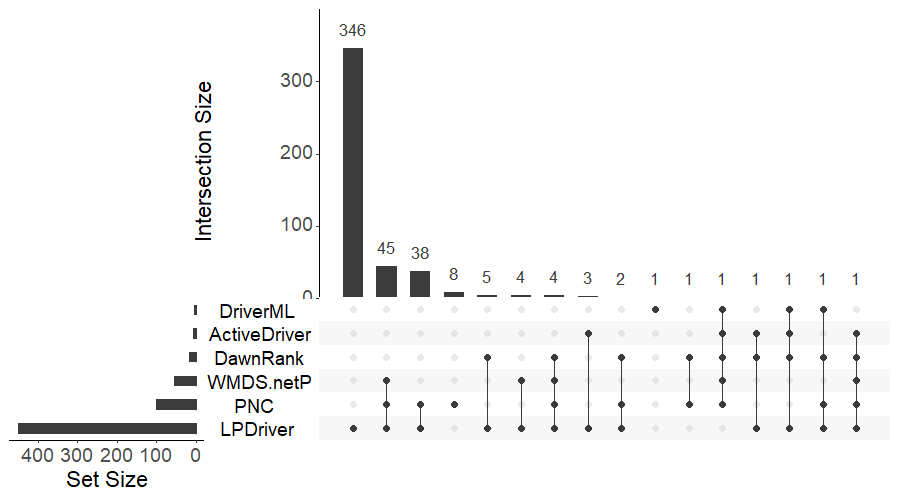


Fig 10. Overlap among different methods for THCA.


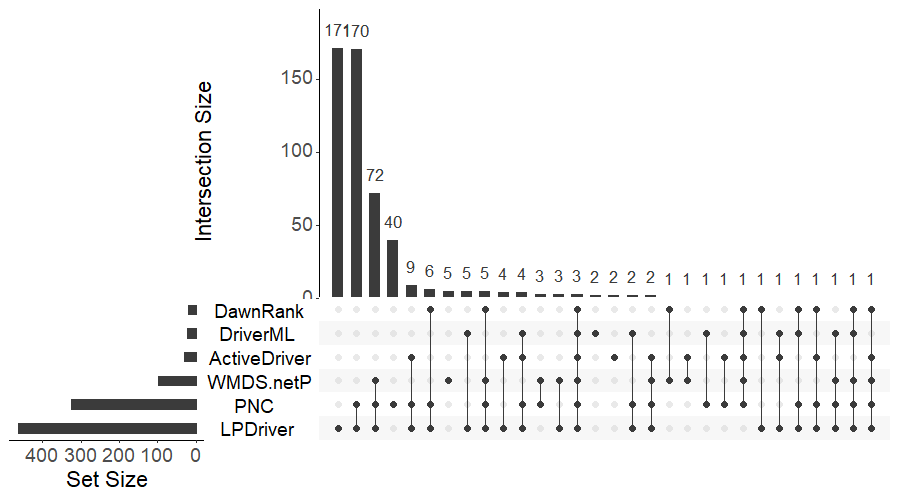


Fig 11. Overlap among different methods for UCEC.
